# Parenting, Adolescent Sensation Seeking, and Subsequent Substance Use: Moderation by Adolescent Temperament

**DOI:** 10.1007/s10964-023-01765-y

**Published:** 2023-03-24

**Authors:** Sabina Kapetanovic, Susannah Zietz, Jennifer E. Lansford, Dario Bacchini, Marc H. Bornstein, Lei Chang, Kirby Deater-Deckard, Laura Di Giunta, Kenneth A. Dodge, Sevtap Gurdal, Paul Oburu, Daranee Junla, Concetta Pastorelli, Ann T. Skinner, Emma Sorbring, Sombat Tapanya, Laurence Steinberg, Liliana Maria Uribe Tirado, Saengduean Yotanyamaneewong, Liane Peña Alampay, Suha M. Al-Hassan

**Affiliations:** 1 University West, Trollhättan, Sweden; 2 Duke University, Durham, NC USA; 3 University of Naples “Federico II”, Naples, Italy; 4 NICHD & UNICEF, New York, NY USA; 5 University of Macau, Zhuhai, China; 6 University of Massachusetts Amherst, Amherst, MA USA; 7 Università di Roma “La Sapienza”, Rome, Italy; 8 Maseno University, Maseno, Kenya; 9 Chiang Mai University, Chiang Mai, Thailand; 10 Temple University, Philadelphia, PA USA; 11 King Abdulaziz University, Jeddah, Saudi Arabia; 12 Universidad de San Buenaventura, Bogotá, Colombia; 13 Ateneo de Manila University, Quezon, Philippines; 14 Hashemite University, Zarqa, Jordan

**Keywords:** Adolescents, Sensation seeking, Parenting, Substance use, Temperament

## Abstract

Although previous research has identified links between parenting and adolescent substance use, little is known about the role of adolescent individual processes, such as sensation seeking, and temperamental tendencies for such links. To test tenets from biopsychosocial models of adolescent risk behavior and differential susceptibility theory, this study investigated longitudinal associations among positive and harsh parenting, adolescent sensation seeking, and substance use and tested whether the indirect associations were moderated by adolescent temperament, including activation control, frustration, sadness, and positive emotions. Longitudinal data reported by adolescents (*n* = 892; 49.66% girls) and their mothers from eight cultural groups when adolescents were ages 12, 13, and 14 were used. A moderated mediation model showed that parenting was related to adolescent substance use, both directly and indirectly, through sensation seeking. Indirect associations were moderated by adolescent temperament. This study advances understanding of the developmental paths between the contextual and individual factors critical for adolescent substance use across a wide range of cultural contexts.

## Introduction

The transition from childhood to adolescence often involves increased risk taking, including substance use such as alcohol, tobacco, and marijuana. Although adolescent experimentation with substances could be seen as developmentally normative, any substance use with early debut (i.e., before the age of 15) is one of the largest risk factors for development of substance use related problems and addiction (Spear, 2015), which is why adolescent substance use continues to be a global problem in need of more attention (Degenhardt et al., 2016). Adolescent substance use may be understood as a product of individual processes, such as development of sensation seeking, as well as environmental processes, such as parenting practices. The increased level of sensation seeking (i.e., tendency to seek novel and intense sensations and taking risks to attain these sensations; Zuckerman, 1979) during adolescence is an individual component that is linked to heightened risk for substance use in adolescence (LaSpada et al., 2020). Positive parenting (i.e., loving and encouraging parenting behaviors) is linked to reduced substance use in adolescence (Crano & Donaldson. 2018), whereas harsh parenting behaviors, including hitting, shaming, and taking away privileges, is linked to increased adolescent substance use (Hinnant et al., 2015). However, individual and environmental factors do not function in isolation from one another. The effect of individual and environmental factors on adolescent substance use may also vary by adolescent temperament (i.e., individual differences in emotional and self-regulation processes) (Belsky et al., 2007). Therefore, the aims of the current study are to investigate longitudinal associations among positive and harsh parenting, adolescent sensation seeking, and subsequent substance use in early to mid-adolescence and to test whether the links among parenting, adolescent sensation seeking, and substance use are moderated by adolescent temperament, including activation and attention control, frustration, sadness, and positive emotions.

### Sensation Seeking and Substance Use during Adolescence

Individual factors, particularly sensation seeking, play a role in adolescents’ engagement in risky behaviors, including substance use. In developmental psychology, sensation seeking is linked to individuals’ risk appraisal with lowered perception of consequences of risk taking, and conceptualized as an individual reward seeking system that can change over time (Zuckerman, 1979), with a developmentally normative increase during adolescence that coincides with increases in risky behaviors (e.g., Steinberg et al., 2018). In fact, using data from 10 cultures around the globe, scholars showed that sensation seeking follows a ∩-shaped curve, steadily increasing during early and late adolescence, peaking around age 19, and declining as adolescents move into adulthood (Steinberg et al., 2018). Given that adolescents’ self-regulatory system is not fully developed, seeking rewards in forms of sensation and thrills increases adolescents’ risk taking, which may be positive, socially accepted or non-harmful, such as joining the cross-country team or protesting for social justice, or negative and potentially harmful, such as substance use (Duell & Steinberg, 2021). Indeed, longitudinal links have been found between greater adolescent sensation seeking and more alcohol use (Hittner & Swickert, 2006), marijuana use (Kaynak et al., 2013), smoking (Hampson et al., 2013), and substance use in general (Meeus et al., 2021), separate from pubertal development and impulsivity (Kong et al., 2013). Moreover, individual changes in sensation seeking are associated with changes in substance use, such that adolescents and young adults with slower decreases of sensation seeking show more substance use over time (Quinn & Harden, 2013). Clearly, heightened sensation seeking is a risk factor for substance use during adolescence.

### Parenting and Substance Use during Adolescence

Adolescent development is not only a product of individual processes but is to a high degree influenced by adolescents’ social environment, including parents. The biopsychosocial model of adolescent risk taking (Sales & Irwin, 2013) posits that adolescent risk behavior should be understood in light of biological, psychosocial, and contextual factors that simultaneously play roles in adolescent engagement in risk behaviors, such as substance use. Although adolescent sensation seeking may be an important predictor of subsequent substance use, adolescents’ social environments, including parents, provide opportunities or reinforcements for adolescents to either engage in or abstain from substance use (Sales & Irwin, 2013). Indeed, loving and encouraging parenting behaviors are linked to positive developmental outcomes (e.g., Leidy et al., 2012). For example, parental monitoring, parent-child relationship quality, parental support, and parental involvement all emerged as longitudinal predictors of both alcohol initiation and levels of later alcohol use and misuse in a meta-analysis of 131 studies (Yap et al., 2017). Parental involvement and parent-adolescent relationship quality were also identified as important predictors of adolescents’ substance use in a meta-analysis of family-based prevention programs for adolescent substance use (Van Ryzin et al., 2016). Higher quality parent-adolescent relationships and more parental involvement are generally related to less adolescent substance use (e.g., Scholes-Balog et al., 2020) whereas dysfunctional parent-child relationships in which children experience harsh physical discipline, rejection, and avoidance from parents are linked to poor adolescent developmental outcomes in a number of domains, including substance use (Rohner & Lansford, 2017). Underlying processes driving the associations between parenting and adolescent substance use are yet to be fully understood.

### Parenting, Sensation Seeking, and Substance Use in Adolescence

According to the review above, it is clear that environmental and individual processes contribute to adolescents’ substance use. As suggested by the biopsychosocial model of adolescent risk taking (Sales & Irwin, 2013), such processes mutually affect each other, and in turn adolescent risk behaviors. In that sense, parents, in their role as children’s most proximal socializing agents, in part shape the development of children’s cognitive processes such as sensation seeking, which in turn affects children’s behavioral outcomes (Soenens et al., 2019). This theory and empirical findings suggest a mediation model in which the association between parenting and adolescent substance use is at least partially mediated by adolescent sensation seeking. Although no studies have investigated a mediation model linking parenting, sensation seeking, and substance use, with sensation seeking as the driving mechanism between parenting and substance use, research studying child cognitive processes suggests that parenting is a critical factor involved in the development of self-regulation, defined as the ability to manage one’s cognition, emotions, and behaviors (Bridgett et al., 2015) and impulse control (Lansford et al., 2017). In general, positive parenting practices such as monitoring children’s activities (Finkenauer et al., 2005) are linked to better long-term self-regulatory processes in adolescents, while harsh parenting practices such as psychological control (Özdemir et al., 2013) and corporal punishment (Lansford et al., 2017) are linked to poorer regulation of impulses over time. If parenting plays a role for development of self-regulation, as these processes are intrinsically linked (Holmes et al., 2016), it is also possible that parenting practices have impact on development of sensation seeking, particularly during adolescence (Pace et al., 2015). With the use of positive parenting strategies, parents can provide behavioral standards for their adolescent children, but also provide them with strategies to overcome their emotional and behavioral challenges and needs (Bariola et al., 2011) and temper the development of sensation seeking. On the other hand, parents who use harsh parenting practices, including corporal punishment, are less likely to be able to provide the guidance that adolescents need, which could aggravate the development of sensation seeking. Their harsh parenting strategies would elicit negative behavior in adolescents, such as substance use (Brody & Ge, 2001), which in turn contributes to parents escalating their harsh behaviors (Roche et al., 2011). Such a pattern contributes to a coercive chain of parent-child interactions that compromises adolescent cognitive and developmental processes (Patterson, 2016). Thus, parenting practices could influence adolescents’ sensation seeking and, in turn, substance use.

### Does One Size Fit All?

The effect of the environment on child development may be stronger for some children than others. For example, one “goodness-of-fit” theoretical model suggests that some children are particularly affected by negative features in the environment (such as experiencing harsh parenting practices) and benefit less from positive features in the environment (diathesis-stress model; Monroe & Simons, 1991). Another model argues that some children are particularly sensitive to positive and promoting features in the environment (such as positive parenting practices), and less sensitive to negative features in the environment (vantage sensitivity model; Pluess & Belsky, 2013). A third theoretical “goodness-of-fit” model suggests that children are likely differentially susceptible to environmental influences, meaning that children with certain individual characteristics may be sensitive to both negative and positive features in the environment (Pluess & Belsky, 2010). These children are adversely affected when exposed to negative features in an environment yet thrive when exposed to positive features in the environment. The effect of the features in the environment on adolescent development may therefore vary depending on adolescent temperament (Belsky et al., 2007). Temperament is defined as rather stable individual differences in emotional, motor, and attentional reactivity and self-regulation processes, such as activation and attention control and negative and positive affect (Rothbart & Derryberry, 1981) and is linked to a number of adolescent outcomes, including substance use (Martel et al., 2009). Specifically, high attention control and positive affect are protective of early onset of substance use, while negative affect and low activation control are linked to early onset of substance use (Wills et al., 2001).

Although child temperament can be a blueprint for development of regulatory skills (Jaffe et al., 2010), parents’ interactions with children can be an important mechanism through which children develop emotional regulatory skills (Bariola et al., 2011). Some children, in particular those with difficult or adventurous temperamental tendencies (Rioux et al., 2016a), such as poor inhibition control and negative affect (Capaldi & Rothbart, 1992), may seem to be more sensitive to parenting practices. When these children experience poor parenting practices, they are inclined to exhibit more substance use over time than their counterparts with easier temperaments (Rioux et al., 2016b). When parenting behaviors are nurturing and warm, children with adventurous temperamental tendencies tend to exhibit more advantageous developmental outcomes over time (Mesman et al., 2009). Although there is criticism to such an idea (Slagt et al., 2016), other studies suggest that children with adventurous temperaments are differentially susceptible to parenting during adolescence (Rioux et al., 2016a). Despite growing research on the moderating effect by temperament on the direct links between parenting and substance use (e.g., Wills et al., 2009), one question that needs more investigation is whether adolescent temperament moderates the indirect processes linking parenting, adolescent sensation seeking, and substance use during early to mid-adolescence.

Another aspect of the “does one size fit all” question is the extent to which findings regarding associations among parenting, adolescent sensation seeking, and substance use generalize across diverse cultural contexts. Each of these constructs, as well as temperament (in terms of activation and attention control, frustration, sadness, and positive emotions), may show mean level differences across cultures. For example, harsh forms of parenting, such as corporal punishment, are used frequently in some cultures but illegal in others (Global Initiative to End All Corporal Punishment of Children, 2023), and positive aspects of parenting such as warmth may be demonstrated differently in different cultural contexts (e.g., by showing physical affection and saying “I love you” versus by preparing favorite foods or supporting a child’s education; Cheah et al., 2015). Temperament also may be operationalized differently in different cultural contexts, and particular measures may show lower reliability in some languages and with some cultural groups than others (Clark et al., 2015). In addition to mean level differences, associations among these constructs may be similar or different across cultural contexts. For example, associations between some aspects of parenting and adolescent adjustment depend on how normative parenting behaviors are in a particular cultural context (Lansford et al., 2018). Thus, the present study investigates the research questions in a diverse sample of parents and adolescents reflecting a range of cultural contexts that contribute to understanding the generalizability of the findings.

## Current Study

**Fig. 1:**
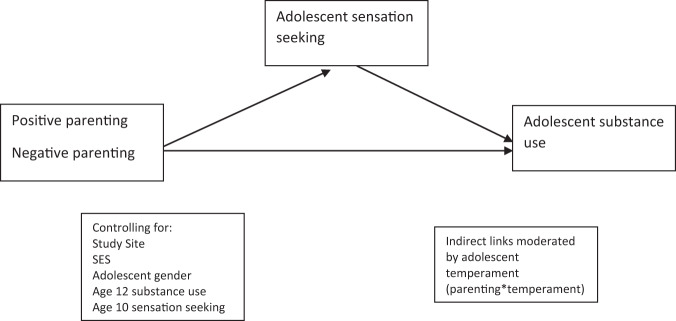
Conceptual mediated moderation model

Based on the review above, the current study will address two important gaps in the adolescent literature. One of those gaps is that it is still unknown whether adolescent sensation seeking, as an individual process that increases during adolescence is a mechanism that drives the associations between parenting and adolescent substance use. Another gap is that literature lacks knowledge of possible moderating effects by adolescent temperament to the possible indirect links among parenting, adolescent sensation seeking, and subsequent substance use. The first aim of this study was to investigate the longitudinal associations among positive and harsh parenting, adolescent sensation seeking, and subsequent substance use in early to mid-adolescence (see Fig. 1). Based on the biopsychosocial model of adolescent risk behavior, it is expected that adolescent sensation seeking will mediate the associations between parenting and adolescent substance use (Hypothesis 1). The second aim was to test whether the indirect effects in aim one were moderated by adolescent temperament (including activation and attention control, frustration, sadness, and positive emotions), or in other words, whether there are significant indirect effects conditioned on adolescent temperament. With the background in “goodness-of-fit” models, it is expected that the indirect effects of parenting on substance use through sensation seeking will be moderated by adolescents’ temperament (Hypothesis 2). As no previous studies have tested the moderation of temperament on indirect effects between parenting and substance use, the direction of effects was only exploratory. These hypotheses were tested in an international sample of families from six countries (China, Colombia, Italy, Philippines, Thailand, and the United States) to enhance generalizability beyond the North American and Western European contexts in which much prior research on parenting, sensation seeking, and adolescent substance use has been conducted to date.

## Methods

### Participants

Participants were from the ongoing Parenting Across Cultures project, a longitudinal sample from 12 groups in 9 countries: Shanghai, China (*n* = 123, 52% girls), Medellín, Colombia (*n* = 108, 56% girls), Naples, Italy (*n* = 102, 52% girls), Rome, Italy (*n* = 111, 50% girls), Zarqa, Jordan (*n* = 114, 47% girls), Kisumu, Kenya (*n* = 100, 60% girls), Manila, Philippines (*n* = 120, 49% girls), Trollhättan/Vänersborg, Sweden (*n* = 129, 48% girls), Chiang Mai, Thailand (*n* = 120, 49% girls), and Durham, North Carolina, United States (*n* = 110 European Americans, 42% girls; *n* = 102 African Americans, 52% girls; *n* = 99 Latinx, 54% girls). Participants were recruited through letters sent home from schools. Parents were asked to sign and return the letter if they were willing to be contacted (in some countries) and contacted by phone to follow up on the letter (in other countries). Children were sampled from schools serving high-, middle-, and low-income families in the approximate proportion to which these income groups were represented in the local population. These sampling procedures resulted in an economically diverse sample that ranged from low income to high income within each site. Data for the present analyses came from waves 5, 7, and 8 of the larger study, when participants were ages 12, 13, and 14, on average, because the measures to address the present research questions were administered at those waves. Age 10 sensation seeking data were included for participants included in the analytical sample. At the eighth year of data collection, 72% of families (*n* = 959) who participated at year 1 continued to provide data, and those who did not provide data at wave 8 did not differ from those who did on parents’ age, parents’ education, or child gender.

### Procedure and Measures

Measures were translated and back-translated and subjected to a process of cultural adaptation to ensure that the measures were linguistically and conceptually equivalent. Measures were administered in Chinese (China), Spanish (Colombia and United States), Italian (Italy), Arabic (Jordan), Dholuo (Kenya), Filipino (Philippines), Swedish (Sweden), Thai (Thailand), and English (United States and Philippines). After parents provided informed consent and children provided assent, interviews were conducted face-to-face, over the telephone, or online. Participants were given modest compensation for their time.

#### Positive parenting age 12

Positive parenting was measured by child report when the children were 12 years of age. The measure consisted of four items rating how much parents engage in positive parenting behaviors such as spending time with their child doing something special that he/she enjoys (Capaldi & Patterson, 1989). Children reported on their mother and father separately. Three of the four items were measured on a 5-point response scale 1 (*never*), 2 (*less than once a month*), 3 (*about once a month*), 4 (*about once a week*), 5 (*almost every day*). The remaining item “How many days a week does your mother/father sit and talk with you?” was measured from 1–7. Because the items were measured on two different response scales, the responses were standardized and averaged. See Appendix 1 for full item list.

#### Harsh parenting age 12

Harsh parenting was measured using four items from the Discipline Interview (Huang et al., 2012; Lansford et al., 2004). Children separately indicated how frequently their mothers and fathers used each of two forms of harsh physical discipline (spank, slap, or hit you; grab or shake you).

#### Sensation seeking ages 10 and 13

Sensation seeking was measured using a subset of six items from the Sensation Seeking Scale (Zuckerman, 1994). Many of the items on the full 19-item scale appear to measure impulsivity (e.g., “I often do things on impulse”). This measure only included the items that clearly indexed thrill- or novelty-seeking (e.g., “I like doing things just for the thrill of it,” see Steinberg et al., 2018). All items were answered as either true or false. See supplemental table for full item list.

#### Adolescent temperament age 13

Early adolescent temperament was measured using mother-report on 22 items from the Early Adolescent Temperament Questionnaire – Revised (EATQ-R; Capaldi & Rothbart, 1992). The subscales included activation control (3 items assessing the capacity to perform an action when there is a strong tendency to avoid it), attention (7 items assessing the capacity to focus attention as well as to shift attention when desired), frustration (6 items assessing negative affect related to interruption of ongoing tasks or goal blocking), sadness (2 items assessing unpleasant affect and lowered mood), and positive emotions (4 items assessing joviality, alertness and positive mood state). See supplemental table for full item list.

#### Substance use age 14

Substance use was measured using one item from the Youth Self Report scale (Achenbach, 1994): “I use alcohol or drugs other than for medical conditions.” The item was measured on a 3-point scale 1 (*not true*), 2 (*somewhat or sometimes true*), 3 (*very true or often true*). Due to low rates on endorsement, response options 2 and 3 were combined.

#### Covariates

Child gender, socioeconomic status (at child age 12), and study site were controlled. Socioeconomic status was measured as a composite of parent-reported mother and father education and family income.

### Analysis Plan

Jordanian, Kenyan, Swedish, and US Latinx participants were not included in the analysis due to no or very low substance use reported among adolescents at age 14. Therefore, the sample population was 892 participants, including 449 (50.34%) boys and 443 (49.66%) girls. Table 1 provides the means, standard deviations, and sample sizes for each of the study sites.

**Table 1:** Means and standard deviations of key variables and percent of sample using substances at 14

	SES	Positive Parenting (age 12)	Harsh Parenting (age 12)	Sensation Seeking (age 10)	Sensation Seeking (age 13)	Activation Control (age 13)	Frustration (age 13)	Sadness (Age 13)	Positive Emotions (age 13)	Substance Use (age 14)
Overall	0.069 (0.90)	−0.04 (0.66)	0.10 (0.57)	−0.01 (0.53)	0.01 (0.56)	−0.04 (0.58)	0.05 (0.59)	0.03 (67)	−0.01 (0.58)	14.42%
China	0.39 (0.66)	−0.07 (0.67)	−0.01 (0.53)	−0.38 (0.51)	−0.35 (0.52)	−0.11 (0.44)	−0.07 (0.37)	0.05 (0.44)	−0.20 (0.59)	4.55%
Colombia	−0.62 (0.87)	0.07 (0.71)	0.21 (0.59)	−0.21 (0.51)	−0.02 (0.57)	−0.11 (0.63)	0.28 (0.74)	0.23 (0.84)	0.15 (0.39)	17.95%
Italy - Naples	−0.48 (0.76)	−0.08 (0.67)	0.15 (0.65)	0.03 (0.54)	0.13 (0.63)	−0.20 (0.71)	0.19 (0.64)	0.08 (0.79)	0.14 (0.45)	16.47%
Italy - Rome	0.04 (0.87)	−0.28 (0.69)	0.21 (0.55)	0.15 (0.55)	0.19 (0.59)	−0.11 (0.60)	0.12 (0.56)	0.06 (0.74)	0.10 (0.58)	30.69%
Philippines	0.07 (0.82)	0.07 (0.53)	0.18 (0.60)	0.11 (0.43)	0.08 (0.43)	−0.03 (0.49)	0.04 (0.55)	0.07 (0.60)	−0.05 (0.51)	5.56%
Thailand	−0.12 (0.78)	−0.19 (0.72)	0.21 (0.63)	0.06 (0.49)	0.10 (0.43)	0.01 (0.54)	−0.18 (0.50)	−0.15 (0.52)	−0.28 (0.67)	12.94%
US - African American	0.16 (0.58)	0.10 (0.63)	−0.09 (0.45)	0.11 (0.49)	−0.08 (0.55)	0.12 (0.65)	−0.05 (0.68)	−0.07 (0.72)	0.06 (0.44)	5.68%
US - European American	1.06 (0.64)	0.12 (0.51)	−0.08 (0.43)	0.11 (0.49)	−0.02 (0.57)	0.11 (0.50)	0.05 (0.52)	−0.01 (0.63)	0.15 (0.40)	14.77%

First an a priori confirmatory factor analysis (CFA) of the latent constructs of harsh parenting at age 12, positive parenting at age 12, sensation seeking at ages 10 and 13, and the five temperament constructs at age 13 (activation control, attention, frustration, sadness, and positive emotions) was conducted using M*plus* version 8 (Muthén & Muthén, 2012). A weighted least squares estimator, which performs well with skewed data such as the binary sensation seeking items, was used (Liang & Yang, 2014). Good model fit is defined by a non-significant chi-square test, RMSEA less than or equal to 0.06, and CFI/TLI greater than or equal to 0.90 (Hu & Bentler, 1999).

Attention control could not be estimated due to poor fit. Therefore, it was not used in further analyses. The original model had an acceptable RMSEA (0.035, 90% CI: [0.033, 038]) and SRMR (0.06), but a low CFI (0.87) and TLI (0.86). Select changes to the CFA model were made using modification indices and tested if the more restricted alternative nested model with the modification is not significantly worse than the original model with more free parameters. Chi-square difference testing was used to ascertain whether dropping items with low loadings did not significantly worsen model fit. The final model moved one item from frustration to sadness, dropped one item from positive emotion, dropped one item from activation control, and correlated the residuals of child report of mother behavior and child report of father behavior in each item in positive parenting and harsh parenting. The final model fit the data well (RMSEA 0.03, 90% CI: [0.27, 0.33]; CFI: 0.91, TLI: 0.90). See supplemental table for CFA results.

To ensure that the above scales were suitable for use in this sample, measurement invariance across the eight cultures was measured using the alignment method (Asparouhov & Muthén, 2014). Muthén and Asparouhov (2014) suggest that approximate measurement invariance is attained if less than 20–25% of parameters are noninvariant. Overall, level of non-invariance for positive parenting (14%), harsh parenting (0%), sensation seeking at age 10 (0%), sensation seeking at age 13 (2%), activation control (6%), frustration (0%), sadness (0%), and positive emotions (4%) fell below the 25% threshold indicating acceptable measurement invariance across groups.

Due to the complexity of the hypothesized model and corresponding concerns about model convergence, factor scores from the CFA were used to estimate the final model. Due to the small number of study sites and the number of respondents within study sites, clustering within study site was adjusted using dummy variables, thus treating the effect of study site as a fixed effect. The index site was Rome, Italy because Rome was the site with a level of sensation seeking closest to the overall average. Given that the dependent variable of substance use was binary, a maximum likelihood estimator with the categorical option was used, as this is appropriate for data with non-normal distributions (Bowen & Guo, 2011). Probit estimates represent the amount of *z*-score change in the outcome for every one-unit change in the predictor.

The mediation model was tested using indirect effects (Preacher et al., 2007). Indirect effects were estimated with bootstrapping using 5000 iterations. Because bootstrapped standard errors cannot be estimated using data that use multiple imputation, the analytical sample of 807 participants excluded 85 participants who were missing data on all predictors (9.5%).

**Fig. 2:**
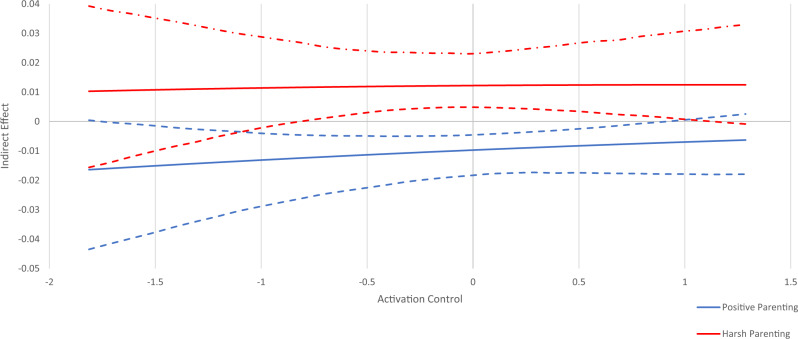
Conditional Indirect Effect of Activation Control. Johnson-Neyman plot of the indirect effects of parenting (positive parenting and harsh parenting) on substance use through sensation seeking conditioned/moderated by the level of activation control. Dashed lines above and below indirect effect indicate high and low 95% confidence intervals. At levels of activation control where the confidence interval does not cross zero, there is a significant indirect effect. At levels of activation control where the confidence interval crosses zero, there is no significant indirect effect

**Fig. 3:**
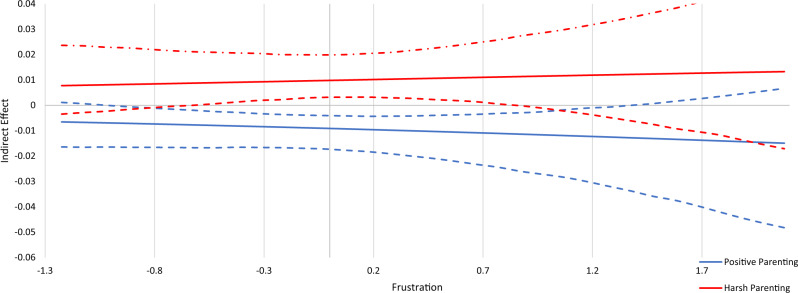
Conditional Indirect Effect of Frustration. Johnson-Neyman plot of the indirect effects of parenting (positive parenting and harsh parenting) on substance use through sensation seeking conditioned/moderated by the level of frustration. Dashed lines above and below indirect effect indicate high and low 95% confidence intervals. At levels of frustration where the confidence interval does not cross zero, there is a significant indirect effect. At levels of frustration where the confidence interval crosses zero, there is no significant indirect effect

**Fig. 4:**
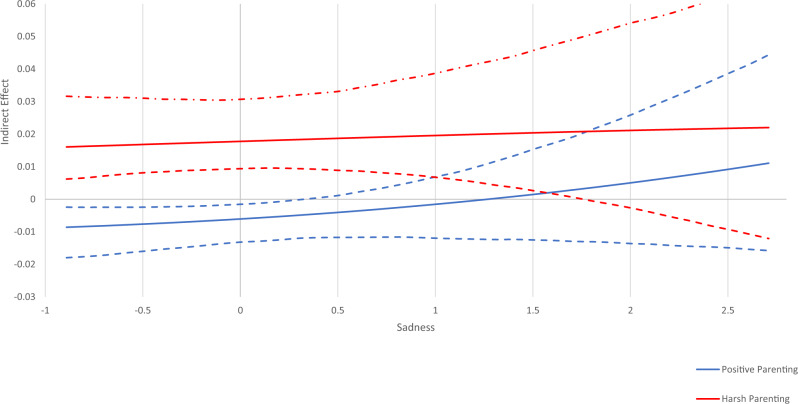
Conditional Indirect Effect of Sadness. Johnson-Neyman plot of the indirect effects of parenting (positive parenting and harsh parenting) on substance use through sensation seeking conditioned/moderated by the level of sadness. Dashed lines above and below indirect effect indicate high and low 95% confidence intervals. At levels of sadness where the confidence interval does not cross zero, there is a significant indirect effect. At levels of sadness where the confidence interval crosses zero, there is no significant indirect effect

**Fig. 5:**
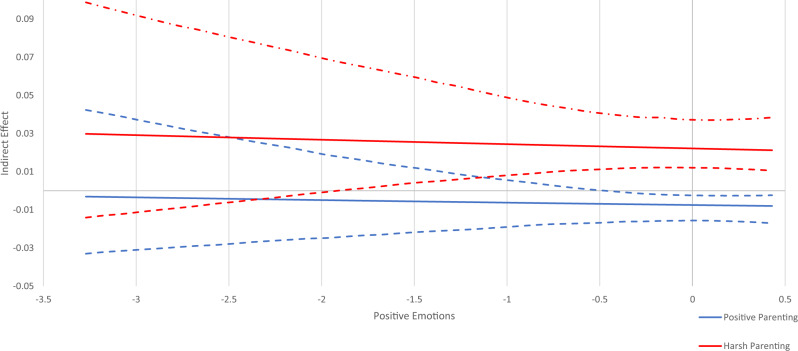
Conditional Indirect Effect of Positive Emotions. Johnson-Neyman plot of the indirect effects of parenting (positive parenting and harsh parenting) on substance use through sensation seeking conditioned/moderated by the level of positive emotions. Dashed lines above and below indirect effect indicate high and low 95% confidence intervals. At levels of positive emotions where the confidence interval does not cross zero, there is a significant indirect effect. At levels of positive emotions where the confidence interval crosses zero, there is no significant indirect effect

To examine the potential moderating role of temperament on the indirect effects, four separate moderated mediation models were run. Controlling for study site, SES, adolescent gender, age 12 substance use and temperament variables, adolescent substance use was regressed on sensation seeking, parenting, and the interaction between parenting and temperament (parenting*temperament). Moreover, sensation seeking was regressed on parenting and the interaction between parenting and temperament. For each temperament variable, the conditional effect was simultaneously estimated on (1) the indirect effect of harsh parenting on substance use through sensation seeking and (2) the indirect effect of positive parenting on substance use through sensation seeking using the model indirect command with the MOD option with bootstrapping using 5000 iterations. The MOD option generates a Johnson-Neyman plot (Figs. 2–5), which depicts the regions of significance of the temperament variables on the total indirect effect.

## Results

### Descriptive Statistics

Table 2 provides the bivariate correlations across the variables for all countries combined. Age 12-positive parenting was negatively correlated with age 14-substance use, while age 12-harsh parenting and age 10 and 13-sensation seeking were positively correlated with age 14-substance use. In addition, low levels of activation control and high levels of frustration and sadness were positively correlated with age 14-substance use. Positive emotion was not significantly correlated with age 14-substance use.

**Table 2:** Correlations between the key variables

	Female	SES	Positive Parenting (age 12)	Harsh Parenting (age 12)	Sensation Seeking (age 10)	Sensation Seeking (age 13)	Activation Control (age 13)	Frustration (age 13)	Depressive Mood (age 13)	Positive Emotions (age 13)
Female	–									
SES	−0.015	–								
Positive Parenting (age 12)	−0.041	0.012	–							
Harsh Parenting (age 12)	−0.041	−0.106*	−0.315**	–						
Sensation Seeking (age 10)	−0.085*	0.069	−0.211**	0.217**	–					
Sensation Seeking (age 13)	−0.025	−0.055	−0.271**	0.315**	0.736**	–				
Activation Control (age 13)	−0.034	0.127**	0.005	−0.235**	−0.013	−0.237**	–			
Frustration (age 13)	0.083*	−0.127**	−0.018	0.219**	−0.034	0.280**	−0.663**	–		
Sadness (age 13)	0.115**	−0.090*	−0.072*	0.072*	−0.203**	0.115**	−0.467**	0.489**	–	
Positive Emotions (age 13)	−0.011	0.043	0.179**	0.123**	0.153**	0.084*	−0.165**	0.064	−0.150**	–
Substance Use (age 14)	−0.006	−0.045	−0.170**	0.117*	0.106*	0.239**	−0.146**	0.156**	0.138**	0.033

Note: * *p* < 0.05 ** *p* < 0.001

### Mediation Analysis

The initial mediation model using factor scores from the CFA did not fit the data well (RMSEA 0.081 90% CI: [0.073, 0.090]; CFI: 0.607, TLI: 0.407, SRMR: 0.114). Select changes were made to the mediation model using modification indices and tested if the more restricted alternative nested model with the modification is not significantly worse than the original model with more free parameters. Nonsignificant covariate paths were pruned. Sensation seeking at age 10 was controlled for living in China (compared to Rome) and correlated age 10-sensation seeking with both age 12-positive parenting and age 12-harsh parenting. The final mediation model fit the data well (RMSEA 0.035, 99% CI: [0.017, 0.053], CFI: 0.971; TLI: 0.939; SRMR: 0.047). Table 3 provides unstandardized and standardized Probit regression coefficients for the mediation model, including bootstrapped confidence intervals, the direct (residual) effects, and the indirect effects of positive parenting and harsh parenting on substance use through sensation seeking.

**Table 3:** Unstandardized and standardized probit regression coefficients for the mediation model

	Unstandardized			Standardized		
	Est.	95% CI		Est	95% CI	
Predictors of Age 14 Substance Use						
Age 12 Substance Use	**1.103**	**0.382**	**1.722**	**0.180**	**0.069**	**0.281**
Age 12 Harsh Parenting	0.000	−0.210	0.203	0.000	−0.110	0.107
Age 12 Positive Parenting	−**0.173**	−**0.347**	−**0.010**	−**0.108**	−**0.217**	−**0.009**
Age 13 Sensation Seeking	**0.499**	**0.233**	**0.737**	**0.267**	**0.123**	**0.399**
Philippines	−**0.715**	−**1.286**	−**0.310**	−**0.230**	−**0.395**	−**0.100**
US African American	−**0.620**	−**1.242**	−**0.233**	−**0.192**	−**0.364**	−**0.074**
Predictors of Age 12 Sensation Seeking						
Age 10 Sensation Seeking	**0.684**	**0.628**	**0.738**	**0.651**	**0.601**	**0.697**
Age 12 Harsh Parenting	**0.136**	**0.084**	**0.182**	**0.137**	**0.087**	**0.184**
Age 12 Positive Parenting	−**0.077**	−**0.122**	−**0.032**	−**0.090**	−**0.143**	−**0.038**
China	−**0.157**	−**0.268**	−**0.069**	−**0.094**	−**0.158**	−**0.042**
US African American	−**0.169**	−**0.295**	−**0.055**	−**0.098**	−**0.171**	−**0.032**
Predictor of Age 10 Sensation Seeking						
China	−**0.418**	−**0.524**	−**0.306**	−**0.263**	−**0.331**	−**0.193**
Effect of Harsh Parenting Through Sensation Seeking						
Indirect Effect	**0.068**	**0.029**	**0.110**	**0.037**	**0.016**	**0.060**
Direct Effect	0.000	−0.210	0.203	0.000	−0.110	0.107
Effect of Positive Parenting Through Sensation Seeking						
Indirect Effect	−**0.039**	−**0.077**	−**0.013**	−**0.024**	−**0.048**	−**0.008**
Direct Effect	−**0.173**	−**0.347**	−**0.010**	−**0.108**	−**0.217**	−**0.009**

Note: Significant confidence intervals are in bold

The direct effect of age 12-positive parenting on age 14-substance use was significant and negative (−0.173, 95% CI: [−0.347, −0.010]). The indirect effect of age 12-positive parenting on age 14-substance use through age 13-sensation seeking was also significant and negative (−0.039, 95% CI: [−0.077, −0.013]), indicating that age 13-sensation seeking partially mediated the relation between age 12-positive parenting and age 14-substance use. Although the direct effect of age 12-harsh parenting on age 14-substance use was not significant (0.000, 95% CI: [−0.210, 0.203]), the indirect effect of age 12-harsh parenting on age 14-substance use through age 13-sensation seeking was significant (0.068, 95% CI: [0.029, 0.110]), indicating that age 13-sensation seeking fully mediated the effect between age 12-harsh parenting and age 14-substance use. Overall, all variables in the mediation analysis explained 56 percent of the variance in age 13-sensation seeking and 22 percent of the variance in age 14 substance-use.

### Moderated Mediation Analysis (Conditional Indirect Effects)

The moderated mediation analyses are depicted in Johnson-Neyman plots in Figs. 2–5. These plots show indirect effects of parenting (positive parenting and harsh parenting) on substance use through sensation seeking conditioned/moderated by levels of the temperament variables. Dashed lines above and below indirect effects indicate high and low 95% confidence intervals. At levels of each temperament variable where the confidence interval does not cross zero, there is a significant indirect effect. At levels of activation control where the confidence interval crosses zero, there is no significant indirect effect.

#### Activation control

The negative indirect effect of positive parenting on substance use through sensation seeking was significant for adolescents with low to average levels of activation control (i.e., from −1.5 to 1), but not for adolescents high in activation control. The positive indirect effect of harsh parenting on substance use through sensation seeking was only significant for adolescents with average activation control (i.e., from −0.8 to 1), but not for adolescents with low and high levels of activation control. This means that adolescents with average levels of activation control are differentially affected by positive and negative parenting through the mediating effect of sensation seeking. In addition, adolescents with low activation control are indirectly affected by positive parenting, but not harsh parenting in terms of adolescent substance use (see Fig. 2).

#### Frustration

The negative indirect effect of positive parenting on substance use through sensation seeking was significant for adolescents with average and slightly above and below average levels of frustration (i.e., from −0.6 to 1.2), but not for adolescents with low and high levels of frustration. The positive indirect effect of harsh parenting on substance use through sensation seeking was only significant for adolescents with average and slightly above and below average frustration (i.e., from −0.5 to 0.8). For adolescents with low and high level of frustration, there was no significant indirect effect of harsh parenting on substance use through sensation seeking. This means, as shown in Fig. 3, that adolescents with average and slightly above and below average levels of frustration are differentially affected by positive and negative parenting.

#### Sadness

The negative indirect effect of positive parenting on substance use through sensation seeking was significant only for adolescents with average and low levels of sadness (i.e., below 0.4), while there was no significant mediation of positive parenting on substance use through sensation seeking for adolescents with above average levels of sadness (i.e., above 0.4). The positive indirect effect of harsh parenting on substance use through sensation seeking was significant for adolescents with low to somewhat higher levels of sadness (i.e., from −0.9 to 1.7). For those with very high levels of sadness, there was no indirect effect of harsh parenting on substance use through sensation seeking. Thus, as shown in Fig. 4, adolescents with low and average levels of sadness are differentially affected by positive and negative parenting. In addition, adolescents with higher levels of sadness are affected by harsh parenting, but not positive parenting in terms of adolescent substance use.

#### Positive emotions

The negative indirect effect of positive parenting on substance use through sensation seeking was significant for adolescents with average and higher levels of positive emotions (i.e., from −0.3 and above). For adolescents with below average levels of positive emotions, there was no indirect effect of positive parenting on substance use through sensation seeking. The positive indirect effect of harsh parenting on substance use through sensation seeking was significant for adolescents with average and low levels of positive emotions (i.e., from −1.8 and above). As shown in Fig. 5, adolescents with average and above average levels of positive emotions are differentially affected by positive and negative parenting. In addition, adolescents with lower levels of positive emotions are affected by harsh parenting but not positive parenting in terms of adolescent substance use.

## Discussion

The biopsychosocial model of adolescent risk behavior (Sales & Irwin, 2013) posits that individual factors such as adolescent sensation seeking, as well as environmental factors, such as parenting, are conjointly involved in the development of adolescent substance use. As an extension to such a theoretical perspective it is also possible that adolescents are differentially affected by environmental cues, such as parenting, based on their temperamental dispositions (Belsky et al., 2007). To address these theoretical ideas, the current study (a) investigated the longitudinal associations among positive and harsh parenting, adolescent sensation seeking, and subsequent substance use in early to mid-adolescence and (b) tested whether the indirect links among parenting, adolescent sensation seeking, and substance use were moderated by adolescent temperament. To assess the generality of these moderated-mediated relations, these relations were assessed in a multi-cultural framework. The results revealed that across multiple cultural contexts, parenting, both directly and indirectly through adolescents’ sensation seeking, is related to adolescent substance use over time and that these links to some extent are moderated by adolescent temperament.

### Links among Parenting, Adolescent Sensation Seeking, and Subsequent Substance Use

Parenting plays an important role in adolescent substance use. Corroborating the results from other studies (e.g., Scholes-Balog et al., 2020), results from the present study indicate that positive parenting (i.e., loving and encouraging parenting behaviors) is predictive of less adolescent substance use. However, parenting is not the sole explanatory factor in terms of adolescent substance use. Adolescent sensation seeking has been shown to be an important mediator of links between parenting and adolescent substance use. Although the effect of positive parenting on adolescent substance use is only partially mediated by adolescent sensation seeking, the effect of harsh parenting (parents grabbing, hitting, and shaming when disciplining their children) is fully mediated by adolescent sensation seeking. According to the biopsychosocial model of adolescent risk behaviors (Sales & Irwin, 2013), adolescent individual and environmental processes simultaneously affect the development of risk behaviors, such as substance use. In this sense, parents, as proximal agents in adolescents’ social environment, would facilitate or inhibit the development of sensation seeking, which in turn would play an important role in terms of adolescent engagement in substance use over time. Indeed, these findings suggest that in different cultural contexts harsh parenting in particular is linked to the development of adolescent sensation seeking which in turn predicts adolescent engagement in substance use.

One explanation for the processes linking parenting, adolescent sensation seeking, and subsequent substance use stems from the idea that parents, as well as others in adolescents’ social systems, socialize children’s rates and expressions of risk behaviors (Soenens et al., 2019). Indeed, parents who put effort into minimizing opportunities for their children to engage in risk behaviors could indirectly influence the development of sensation seeking in their children. Parents who are involved in their children’s lives could create healthy venues for their children’s needs to explore and experience novelties and in such a way change how their sensation seeking is expressed. This, in turn, would minimize the risk of poor behavioral outcomes such as substance use. By contrast, experiencing shaming and physical discipline from parents puts a strain on parent-child bonds and children’s willingness to spend time with parents (Rohner & Lansford, 2017), particularly in cultural contexts where physical discipline is not a norm (Lansford et al., 2018). Children could experience heightened need for thrills and novelties which, without proper guidance, could be expressed in risk behaviors such as substance use.

Another explanation for the processes linking parenting, adolescent sensation seeking, and subsequent substance use concerns the imbalance in the development of cognitive control. Adolescent sensation seeking is linked to risk proneness, and is to some extent separated from the process of development of cognitive control that inhibits impulsive and reckless behavior (Meeus et al., 2021). Although studies on the predictors of sensation seeking are scarce, research on adolescent cognitive processes suggests that the development of adolescent self-regulatory skills, thus managing one’s impulses and behaviors, is at least in part explained by particular parenting strategies (Morawska et al., 2019). In the context of positive and nurturing parenting, children have opportunities to learn to accept their emotions and to handle behavioral difficulties. However, in the context of harsh and unpredictable parenting environments, parents may respond to children inconsistently or inadequately, which in turn would provide children with fewer opportunities to test, learn, and internalize self-regulatory skills. Although this specific process could not be tested in the current study, it is possible that an increase of self-regulatory skills and cognitive control attenuates the development of sensation seeking in adolescence which in turn minimizes the risk of adolescent engagement in substance use. Indeed, Meeus and colleagues (2021) have shown that there is substantial heterogeneity in the development of cognitive control and sensation seeking in adolescence. Although some adolescents experience an imbalance in these two neurobiological processes favoring sensation seeking, for some adolescents, the development of cognitive control is stronger than the development of sensation seeking, which in turn is related to less engagement in substance use. How parenting contributes to heterogeneity in development of these neurobiological processes is a topic for future exploration.

### Does Adolescent Temperament Play a Role?

Adolescents can react differently to influences in their social environment. Individual differences in temperament may play an important role in how adolescents respond to parenting practices (Belsky et al., 2007). A series of mediation models moderated by child activation control, frustration, sadness, and positive emotion, showed that child temperament moderated the indirect links among parenting, adolescent sensation seeking, and subsequent substance use. Specifically, positive parenting was negatively and indirectly, through sensation seeking, related to subsequent substance use in adolescents with low to average levels of activation control; in adolescents with average and slightly above and below average levels of frustration; in adolescents with average and low levels of sadness; and in adolescents with average and higher levels of positive emotions. Harsh parenting was positively and indirectly, through sensation seeking, related to subsequent substance use in adolescents with average levels of activation control; in adolescents with average and slightly above and below average levels of frustration; in adolescents with low to somewhat higher levels of sadness; and in adolescents with average and low levels of positive emotions. These findings indicate that adolescents with average levels of activation control, adolescents with average and slightly above and below average levels of frustration, adolescents with low to average levels of sadness, and adolescents with average and above average levels of positive emotions were differentially affected by positive and harsh parenting.

According to differential susceptibility theory (e.g., Belsky & Pluess, 2009), certain individual characteristics can be disadvantageous in adverse environments but advantageous in the context of enriching environments. That means that children who are vulnerable to adversity could also be plastic in the sense that they could be particularly susceptible to the benefits of supportive and positive environments. While these children would struggle in an adverse home environment, the same children would thrive in an advantageous home environment. Expanding the previous literature testing differential susceptibility theory in adolescents (e.g., Rioux et al., 2016a), the present study showed that adolescent temperament plays a role in the joint social and individual processes in the development of adolescent substance use. For adolescents with average levels of activation control, frustration, sadness, and positive emotions, parental practices are related to the development of certain individual processes, such as adolescent sensation seeking, which in turn has bearing on development of adolescent substance use. Specifically, adolescents who to some extent can control their behaviors and feelings and those who approach situations with optimism and positivity could, in comparison to their counterparts, be more open to parents’ attempts to be involved in their lives. Parents could then provide guidance, norms, and behavioral outlets that may discourage the development of sensation seeking and subsequent risk behaviors such as substance use. At the same time, these adolescents would also be vulnerable to parents’ harsh parenting practices, which through intensification of thrill seeking, would make them more liable to search for emotional outlets, such as substance use. Although individuals seem to be the most susceptible to environmental cues during early childhood (Slagt et al., 2016), it is critical that adolescent temperamental tendencies and parenting practices are attuned in order for adolescents to have more optimal development.

Although some adolescents seem to be more susceptible to both positive and negative parenting (Belsky et al., 2007), other adolescents seem to be more sensitive to positive parenting practices but more resilient to harsh parenting as suggested by the vantage sensitivity theoretical model (Pluess & Belsky, 2013). The present results showed that adolescents with low activation control seem to benefit from positive parental practices which indirectly, through sensation seeking, have impact on adolescent subsequent substance use. On the other hand, these adolescents may be more resilient to parental harsh practices. Parents’ support and care seem to be of particular importance for regulation of sensation seeking and in turn substance use in adolescents with difficulties regulating their behavior. Indeed, poor behavioral regulation is a well-established risk for substance use in adolescence (Kim-Spoon et al., 2016). Given that sensation seeking increases in adolescence as a normative developmental process (Steinberg et al., 2018), adolescents with poor regulatory skills would be of particular need for adequate support and guidance from parents in order to learn to self-regulate and abstain from risky behaviors such as substance use. As findings from the current study suggest, when parents are warm and involved in their children’s lives, adolescents with poor behavioral regulation have more opportunities to learn to regulate their sensation seeking tendencies and in turn be protected from involvement in substance use.

In addition, the results also support the diathesis-stress hypothesis (Pluess & Belsky, 2010), suggesting that adolescents high in sadness and adolescents with low levels of positive emotions are particularly sensitive to harsh parenting, while rather resistant to positive parenting in terms of adolescent substance use. These somewhat conflicting findings indicate that adolescents with poor emotional regulatory skills may be vulnerable to harsh parenting practices in a sense that shaming and physical discipline from parents intensify development of adolescent sensation seeking and in turn subsequent substance use. Although parents and children mutually affect each other in terms of their emotional regulation (Kiel & Kalomiris, 2015), parents, through their behaviors and emotional reactions, are models through which adolescents learn strategies to regulate their emotions and in turn their behaviors (Bariola et al., 2011). When relationships with parents are strained, adolescents with low mood and negative appraisal tendencies may particularly have difficulties in learning to regulate their emotions, which in turn would be disadvantageous in terms of development of sensation seeking and subsequent substance use.

The results of the current study provide new theoretical and practical insight about the processes linking parenting and adolescent individual characteristics with adolescent substance use. Complex models where positive and harmful environmental factors and individual processes are at interplay, help to identify the specificity of the results and lessen the risk of drawing incorrect inferences about the extent to which findings generalize to all adolescents, regardless of their personal traits or ecological contexts. As shown in this study, different parenting practices have different impact on adolescent psychosocial development, depending on the temperamental tendencies of the adolescents and their interactions with parents. Moreover, the findings may help parents as well as professionals in health care and social services to identify the mechanisms critical for development of substance use, and to understand to what extent parental practices could alleviate or intensify the development of sensation and thrill seeking and for whom, knowledge that could be used for adolescent substance use prevention. In that sense, substance use and parenting interventions should be extended with more emphasis on adolescent individual characteristics and their interaction with parenting practices. Expecting that all adolescents are equally affected by different parenting practices may be faulty, or even harmful for adolescent development, and may devalue the trust in the expertise that professionals who work with parents and adolescents hold. Instead, personalized parenting and care approaches would help parents, health care, and social services move toward efficacy in their efforts and their understanding of adolescents’ specific needs (Belsky, 2016). In other words, parenting interventions could be tailored to focus on not only what parents should do, but also focus on *how* and for *which* adolescents specific parenting practices may be beneficial or harmful in terms of adolescent substance use. Parenting should be tailored to children’s unique individual characteristics and needs in order for children to have the best possible developmental outcomes. When parenting practices are attuned with adolescent unique needs, parents are more likely to provide guidance to their adolescents, potentially yielding more positive developmental outcomes in adolescents.

### Strengths and Limitations

Although this study has some important strengths, such as a cross-cultural sample followed longitudinally with parent and adolescent reports, there are some limitations that need to be mentioned. The measure for substance use was only one item that did not delineate use of different types of substances or severity of use. Although alcohol use is likely to be the most prevalent substance used during adolescence (in comparison to other drugs), inferences on the specificity of substance use cannot be made. However, the use of any substances, including alcohol and marijuana, is illegal among adolescents younger than 18 at all study sites, which makes the implications of the study of importance for the parenting literature and beyond. We also focused on sensation seeking in the present study but acknowledge the need for future research to include related constructs, such as impulsivity. Additionally, due to low variability, the substance use measure was collapsed to “any” versus “no” use, which is why differentiation between frequency among users was not possible. To address sparse data bootstrap confidence intervals that are robust to sparse data were used (Lee et al., 2021). Moreover, adolescent temperament variable was measured at participant age 13, a period of pubertal development. Although emotional reactivity may intensify during this developmental period (Mendle, 2014), temperament is generally considered to be a stable trait (Shiner et al., 2012). The parenting measures and sensation seeking were collected during different waves, which is why the processes between parenting and sensation seeking in a bidirectional manner could not be tested. As adolescent development is dynamic and interrelated with the context (Sameroff, 2010), future research should pay more attention to bidirectional links in associations among parenting, adolescent sensation seeking, and substance use. Finally, due to poor fit, the attention control sub-scale of the temperament measure across the sample could not be used. Additionally, minor changes were made to the temperament sub-scales to order to improve model fit. These changes were conceptually justified based on the items in question.

The role of parenting for adolescent adjustment seems to be universal, although differences in the parenting norms as well as behavioral expectations may differ both between and within cultures (Lansford et al., 2018). Although substance use is illegal in all the cultural contexts included in the current study, corporal punishment is not (Global Initiative to End All Corporal Punishment of Children, 2023). Parenting norms as well as norms about normative behavioral development in adolescents could have bearing for the effect of parenting on adolescent psychosocial development (Lansford et al., 2018). In what way parenting norms play role for the interplay between environmental and individual processes is a topic for further research. Finally, although the samples were designed to be representative of the cities from which they were drawn, they are not nationally representative, so the study findings may not generalize to entire countries included in this study.

## Conclusion

An important gap in the literature has been the lack of research on the specific processes linking parenting, adolescent sensation seeking, and subsequent substance use as well as the role that adolescent temperament characteristics may play in the associations among these links. This study advances understanding of the developmental paths between the contextual and individual factors critical for adolescent substance use across a wide range of cultural contexts. Specifically, positive parenting practices, including spending quality time with children, is protective against adolescent substance use, whereas parents’ harsh practices, including shaming and physical discipline, have adverse effects on adolescent substance use across cultures. Positive parenting practices seem to inhibit development of sensation seeking, but harsh parenting seems to facilitate the development of adolescent sensation and thrill seeking, which subsequently predicts adolescent substance use. Moreover, adolescents with average levels of activation control, frustration, sadness, and positive emotions are differentially sensitive to parenting practices. These adolescents fare better in the context of positive parenting, but they seem to fare worse in the context of harsh and unstable parenting environments. Other adolescents, such as those with low activation control, seem to be more affected by positive parenting than harsh parenting in terms of their sensations seeking and subsequent substance use. In contrast, adolescents with temperamental tendencies of high sadness and low positive emotions are more affected by harsh parenting than positive parenting in terms of sensation seeking and subsequent substance use. Taken together, these findings suggest the importance of taking into account adolescents’ temperamental characteristics in understanding how parenting and sensation seeking are related to adolescents’ substance use.

## Appendix 1. Confirmatory Factor Analysis Results

**Table:**

Item	Standardized loading	S.E.
**Positive Parenting**		
How often does your father notice when you are doing a good job and let you know	0.704***	0.029
How many days a week does your father sit and talk with you	0.484***	0.042
How much time do you spend with your father doing something special that you enjoy	0.579***	0.034
How often does your father show you he likes it when you help around the house	0.611***	0.034
How often does your mother notice when you are doing a good job and let you know	0.397***	0.041
How many days a week does your mother sit and talk with you	0.588***	0.030
How much time do you spend with your mother doing something special that you enjoy	0.673***	0.028
How often does your mother show you she likes it when you help around the house	0.590***	0.032
**Harsh Parenting**		
How frequently does your mother and/or mother spank, slap, or hit you?	0.764***	0.065
How frequently does your father and/or mother spank, slap, or hit you?	0.709***	0.063
How frequently does your mother grab or shake you?	0.811***	0.073
How frequently does your father grab or shake you?	0.875***	0.076
**Age 13 Sensation Seeking**		
I sometimes do “crazy” things just for fun	0.764***	0.045
I like to have new and exciting experiences and feelings even if they are a little frightening	0.538***	0.059
I like doing things just for the thrill of it	0.676***	0.048
I sometimes like to do things that are a little frightening	0.637***	0.050
I’ll try anything once	0.474***	0.056
I like wild and “crazy” parties	0.593***	0.049
**Age 10 Sensation Seeking**		
I sometimes do “crazy” things just for fun	0.715***	0.048
I like to have new and exciting experiences and feelings even if they are a little frightening	0.518***	0.058
I like doing things just for the thrill of it	0.504***	0.053
I sometimes like to do things that are a little frightening	0.700***	0.049
I’ll try anything once	0.273***	0.059
I like wild and “crazy” parties	0.645***	0.049
**Adolescent Temperament**		
*Frustration*		
Your son /daughter get very irritated when someone criticizes her/him	0.608***	0.032
Hates it when people don’t agree with him/her	0.654***	0.028
Gets mad when even mildly criticized	0.749***	0.022
Gets angry when s/he can’t find something s/he wants	0.685***	0.028
Feels like crying over very little on some days	0.549***	0.033
*Sadness*		
Often does not seem to enjoy things as much as his/her friends	0.728***	0.027
Is sad more often than other people realize	0.744***	0.024
Sometimes seems sad even when s/he should be enjoying her/himself	0.808***	0.025
*Positive Emotions*		
Often feels gratified when s/he accomplishes a goal	0.916***	0.015
Often feels joy when good things happen to him/her	0.800***	0.016
Often feels proud when s/he finishes a difficult task	0.749***	0.018
*Activation Control*		
Opens presents before s/he is supposed to	0.605***	0.044
Has a hard time waiting for his/her turn to speak when excited	0.714***	0.049

Note: ** p < 0.001
